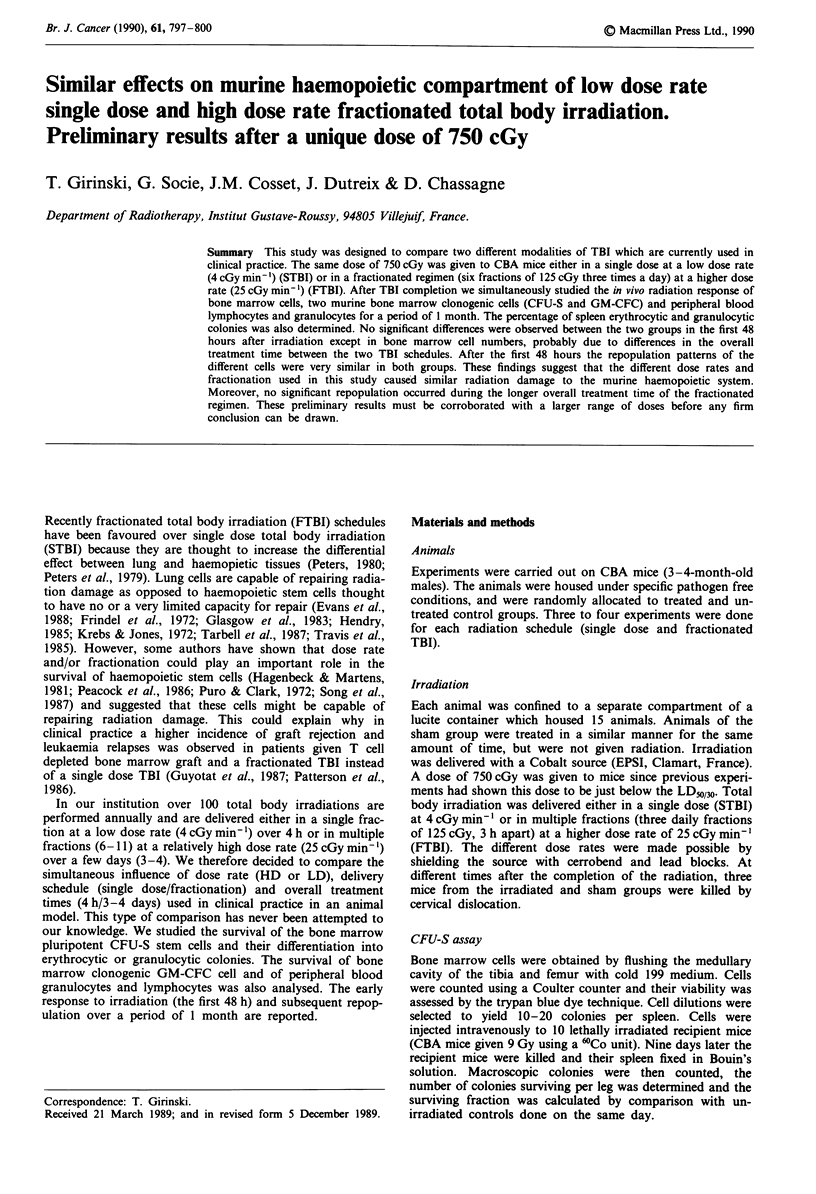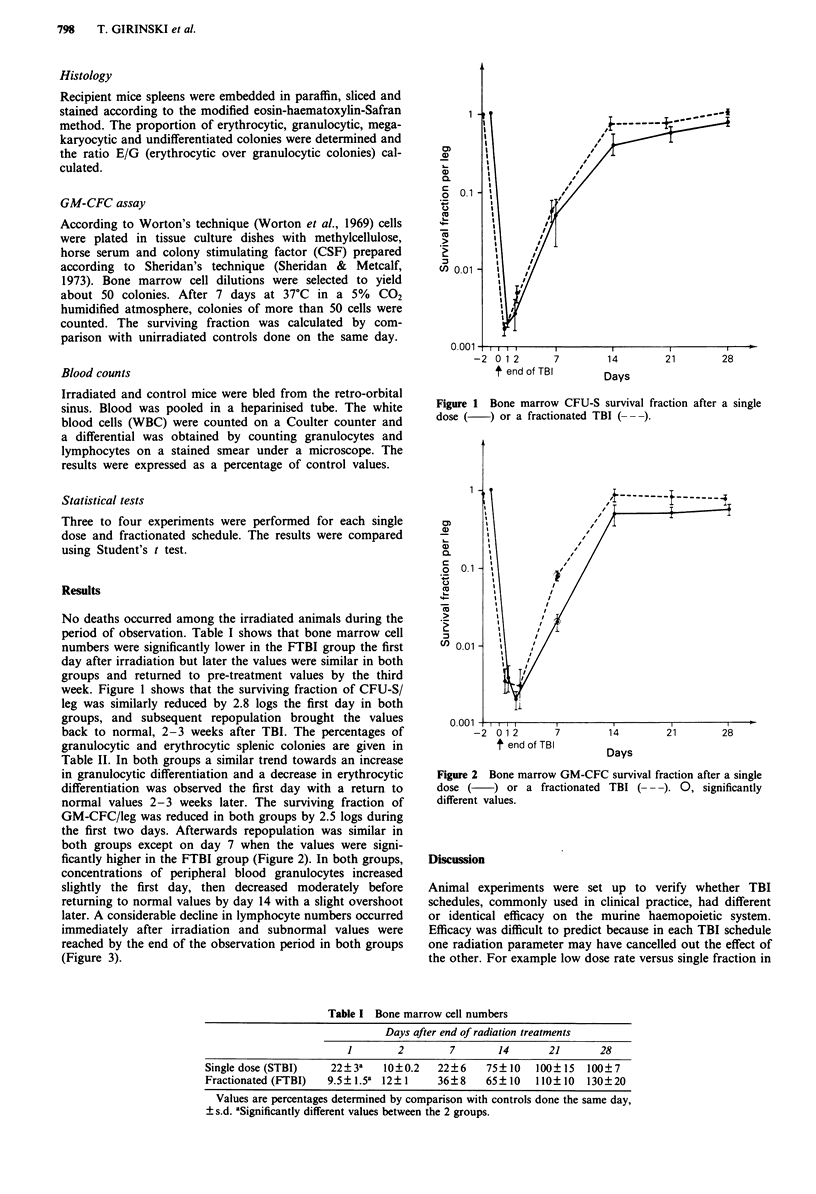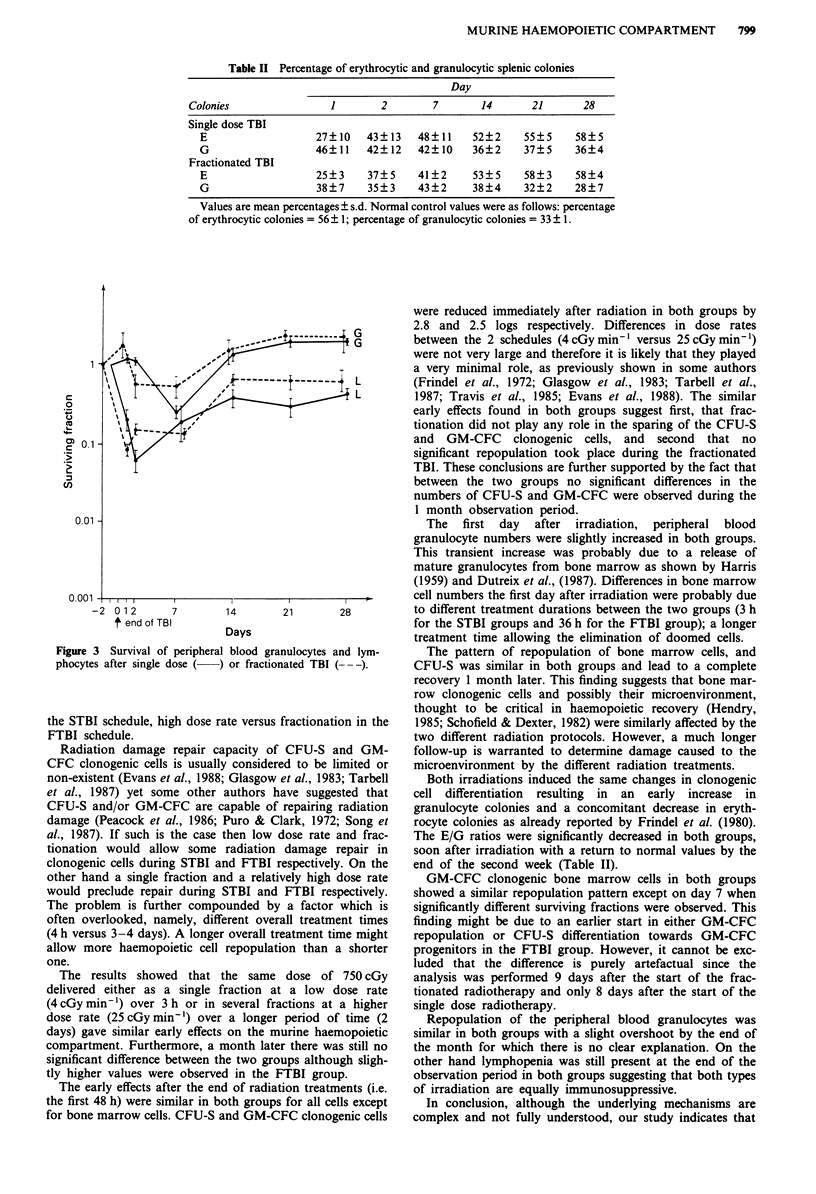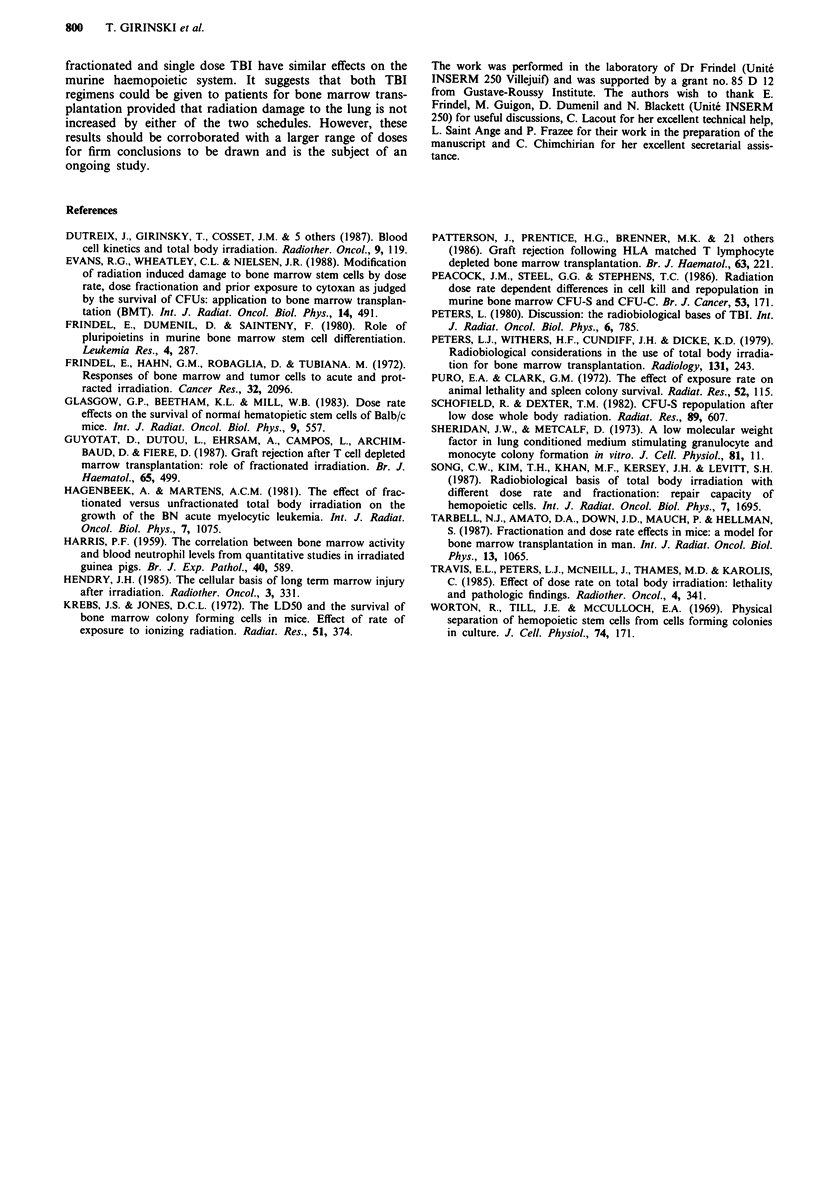# Similar effects on murine haemopoietic compartment of low dose rate single dose and high dose rate fractionated total body irradiation. Preliminary results after a unique dose of 750 cGy.

**DOI:** 10.1038/bjc.1990.180

**Published:** 1990-06

**Authors:** T. Girinski, G. Socie, J. M. Cosset, J. Dutreix, D. Chassagne

**Affiliations:** Department of Radiotherapy, Institut Gustave-Roussy, Villejuif, France.

## Abstract

This study was designed to compare two different modalities of TBI which are currently used in clinical practice. The same dose of 750 cGy was given to CBA mice either in a single dose at a low dose rate (4 cGy min-1) (STBI) or in a fractionated regimen (six fractions of 125 cGy three times a day) at a higher dose rate (25 cGy min-1) (FTBI). After TBI completion we simultaneously studied the in vivo radiation response of bone marrow cells, two murine bone marrow clonogenic cells (CFU-S and GM-CFC) and peripheral blood lymphocytes and granulocytes for a period of 1 month. The percentage of spleen erythrocytic and granulocytic colonies was also determined. No significant differences were observed between the two groups in the first 48 hours after irradiation except in bone marrow cell numbers, probably due to differences in the overall treatment time between the two TBI schedules. After the first 48 hours the repopulation patterns of the different cells were very similar in both groups. These findings suggest that the different dose rates and fractionation used in this study caused similar radiation damage to the murine haemopoietic system. Moreover, no significant repopulation occurred during the longer overall treatment time of the fractionated regimen. These preliminary results must be corroborated with a larger range of doses before any firm conclusion can be drawn.


Br. J. Cancer (1990), 61, 797-800 © Macmillan Press Ltd., 1990

### Similar effects on murine haemopoietic compartment of low dose rate single dose and high dose rate fractionated total body irradiation. Preliminary results after a unique dose of 750 cGy

T. Girinski, G. Socie, J.M. Cosset, J. Dutreix & D. Chassagne

*Department of Radiotherapy, Institut Gustave-Roussy, 94805 Villejuif, France.*

**Summary** This study was designed to compare two different modalities of TBI which are currently used in clinical practice. The same dose of 750 cGy was given to CBA mice either in a single dose at a low dose rate (4 cGy $min^{-1}$) (STBI) or in a fractionated regimen (six fractions of 125 cGy three times a day) at a higher dose rate (25 cGy $min^{-1}$) (FTBI). After TBI completion we simultaneously studied the *in vivo* radiation response of bone marrow cells, two murine bone marrow clonogenic cells (CFU-S and GM-CFC) and peripheral blood lymphocytes and granulocytes for a period of 1 month. The percentage of spleen erythrocytic and granulocytic colonies was also determined. No significant differences were observed between the two groups in the first 48 hours after irradiation except in bone marrow cell numbers, probably due to differences in the overall treatment time between the two TBI schedules. After the first 48 hours the repopulation patterns of the different cells were very similar in both groups. These findings suggest that the different dose rates and fractionation used in this study caused similar radiation damage to the murine haemopoietic system. Moreover, no significant repopulation occurred during the longer overall treatment time of the fractionated regimen. These preliminary results must be corroborated with a larger range of doses before any firm conclusion can be drawn.

Recently fractionated total body irradiation (FTBI) schedules have been favoured over single dose total body irradiation (STBI) because they are thought to increase the differential effect between lung and haemopietic tissues (Peters, 1980; Peters *et al.*, 1979). Lung cells are capable of repairing radiation damage as opposed to haemopoietic stem cells thought to have no or a very limited capacity for repair (Evans *et al.*, 1988; Frindel *et al.*, 1972; Glasgow *et al.*, 1983; Hendry, 1985; Krebs & Jones, 1972; Tarbell *et al.*, 1987; Travis *et al.*, 1985). However, some authors have shown that dose rate and/or fractionation could play an important role in the survival of haemopoietic stem cells (Hagenbeck & Martens, 1981; Peacock *et al.*, 1986; Puro & Clark, 1972; Song *et al.*, 1987) and suggested that these cells might be capable of repairing radiation damage. This could explain why in clinical practice a higher incidence of graft rejection and leukaemia relapses was observed in patients given T cell depleted bone marrow graft and a fractionated TBI instead of a single dose TBI (Guyotat *et al.*, 1987; Patterson *et al.*, 1986).

In our institution over 100 total body irradiations are performed annually and are delivered either in a single fraction at a low dose rate (4 cGy $min^{-1}$) over 4 h or in multiple fractions (6-11) at a relatively high dose rate (25 cGy $min^{-1}$) over a few days (3-4). We therefore decided to compare the simultaneous influence of dose rate (HD or LD), delivery schedule (single dose/fractionation) and overall treatment times (4 h/3-4 days) used in clinical practice in an animal model. This type of comparison has never been attempted to our knowledge. We studied the survival of the bone marrow pluripotent CFU-S stem cells and their differentiation into erythrocytic or granulocytic colonies. The survival of bone marrow clonogenic GM-CFC cell and of peripheral blood granulocytes and lymphocytes was also analysed. The early response to irradiation (the first 48 h) and subsequent repopulation over a period of 1 month are reported.

Correspondence: T. Girinski.
Received 21 March 1989; and in revised form 5 December 1989.

### Materials and methods

### *Animals*

Experiments were carried out on CBA mice (3-4-month-old males). The animals were housed under specific pathogen free conditions, and were randomly allocated to treated and untreated control groups. Three to four experiments were done for each radiation schedule (single dose and fractionated TBI).

### *Irradiation*

Each animal was confined to a separate compartment of a lucite container which housed 15 animals. Animals of the sham group were treated in a similar manner for the same amount of time, but were not given radiation. Irradiation was delivered with a Cobalt source (EPSI, Clamart, France). A dose of 750 cGy was given to mice since previous experiments had shown this dose to be just below the $LD_{50/30}$. Total body irradiation was delivered either in a single dose (STBI) at 4 cGy $min^{-1}$ or in multiple fractions (three daily fractions of 125 cGy, 3 h apart) at a higher dose rate of 25 cGy $min^{-1}$ (FTBI). The different dose rates were made possible by shielding the source with cerrobend and lead blocks. At different times after the completion of the radiation, three mice from the irradiated and sham groups were killed by cervical dislocation.

### *CFU-S assay*

Bone marrow cells were obtained by flushing the medullary cavity of the tibia and femur with cold 199 medium. Cells were counted using a Coulter counter and their viability was assessed by the trypan blue dye technique. Cell dilutions were selected to yield 10-20 colonies per spleen. Cells were injected intravenously to 10 lethally irradiated recipient mice (CBA mice given 9 Gy using a $^{60}Co$ unit). Nine days later the recipient mice were killed and their spleen fixed in Bouin's solution. Macroscopic colonies were then counted, the number of colonies surviving per leg was determined and the surviving fraction was calculated by comparison with unirradiated controls done on the same day.

*Histology*

Recipient mice spleens were embedded in paraffin, sliced and stained according to the modified eosin-haematoxylin-Safran method. The proportion of erythrocytic, granulocytic, megakaryocytic and undifferentiated colonies were determined and the ratio E/G (erythrocytic over granulocytic colonies) calculated.

*GM-CFC assay*

According to Worton's technique (Worton *et al.*, 1969) cells were plated in tissue culture dishes with methylcellulose, horse serum and colony stimulating factor (CSF) prepared according to Sheridan's technique (Sheridan & Metcalf, 1973). Bone marrow cell dilutions were selected to yield about 50 colonies. After 7 days at 37°C in a 5% $CO_2$ humidified atmosphere, colonies of more than 50 cells were counted. The surviving fraction was calculated by comparison with unirradiated controls done on the same day.

*Blood counts*

Irradiated and control mice were bled from the retro-orbital sinus. Blood was pooled in a heparinised tube. The white blood cells (WBC) were counted on a Coulter counter and a differential was obtained by counting granulocytes and lymphocytes on a stained smear under a microscope. The results were expressed as a percentage of control values.

*Statistical tests*

Three to four experiments were performed for each single dose and fractionated schedule. The results were compared using Student's *t* test.

### Results

No deaths occurred among the irradiated animals during the period of observation. Table I shows that bone marrow cell numbers were significantly lower in the FTBI group the first day after irradiation but later the values were similar in both groups and returned to pre-treatment values by the third week. Figure 1 shows that the surviving fraction of CFU-S/leg was similarly reduced by 2.8 logs the first day in both groups, and subsequent repopulation brought the values back to normal, 2–3 weeks after TBI. The percentages of granulocytic and erythrocytic splenic colonies are given in Table II. In both groups a similar trend towards an increase in granulocytic differentiation and a decrease in erythrocytic differentiation was observed the first day with a return to normal values 2–3 weeks later. The surviving fraction of GM-CFC/leg was reduced in both groups by 2.5 logs during the first two days. Afterwards repopulation was similar in both groups except on day 7 when the values were significantly higher in the FTBI group (Figure 2). In both groups, concentrations of peripheral blood granulocytes increased slightly the first day, then decreased moderately before returning to normal values by day 14 with a slight overshoot later. A considerable decline in lymphocyte numbers occurred immediately after irradiation and subnormal values were reached by the end of the observation period in both groups (Figure 3).

**Figure 1** Bone marrow CFU-S survival fraction after a single dose (——) or a fractionated TBI (– – –).

**Figure 2** Bone marrow GM-CFC survival fraction after a single dose (——) or a fractionated TBI (– – –). O, significantly different values.

### Discussion

Animal experiments were set up to verify whether TBI schedules, commonly used in clinical practice, had different or identical efficacy on the murine haemopoietic system. Efficacy was difficult to predict because in each TBI schedule one radiation parameter may have cancelled out the effect of the other. For example low dose rate versus single fraction in

**Table I** Bone marrow cell numbers

| | Days after end of radiation treatments | | | | | |
|---|---|---|---|---|---|---|
| | *1* | *2* | *7* | *14* | *21* | *28* |
| Single dose (STBI) | 22±3[a] | 10±0.2 | 22±6 | 75±10 | 100±15 | 100±7 |
| Fractionated (FTBI) | 9.5±1.5[a] | 12±1 | 36±8 | 65±10 | 110±10 | 130±20 |

Values are percentages determined by comparison with controls done the same day, ±s.d. [a]Significantly different values between the 2 groups.

**Table II** Percentage of erythrocytic and granulocytic splenic colonies

| Colonies | Day 1 | 2 | 7 | 14 | 21 | 28 |
|---|---|---|---|---|---|---|
| Single dose TBI | | | | | | |
| E | 27±10 | 43±13 | 48±11 | 52±2 | 55±5 | 58±5 |
| G | 46±11 | 42±12 | 42±10 | 36±2 | 37±5 | 36±4 |
| Fractionated TBI | | | | | | |
| E | 25±3 | 37±5 | 41±2 | 53±5 | 58±3 | 58±4 |
| G | 38±7 | 35±3 | 43±2 | 38±4 | 32±2 | 28±7 |

Values are mean percentages ± s.d. Normal control values were as follows: percentage of erythrocytic colonies = 56 ± 1; percentage of granulocytic colonies = 33 ± 1.

**Figure 3** Survival of peripheral blood granulocytes and lymphocytes after single dose (——) or fractionated TBI (– – –).

the STBI schedule, high dose rate versus fractionation in the FTBI schedule.

Radiation damage repair capacity of CFU-S and GM-CFC clonogenic cells is usually considered to be limited or non-existent (Evans *et al.*, 1988; Glasgow *et al.*, 1983; Tarbell *et al.*, 1987) yet some other authors have suggested that CFU-S and/or GM-CFC are capable of repairing radiation damage (Peacock *et al.*, 1986; Puro & Clark, 1972; Song *et al.*, 1987). If such is the case then low dose rate and fractionation would allow some radiation damage repair in clonogenic cells during STBI and FTBI respectively. On the other hand a single fraction and a relatively high dose rate would preclude repair during STBI and FTBI respectively. The problem is further compounded by a factor which is often overlooked, namely, different overall treatment times (4 h versus 3–4 days). A longer overall treatment time might allow more haemopoietic cell repopulation than a shorter one.

The results showed that the same dose of 750 cGy delivered either as a single fraction at a low dose rate (4 cGy min$^{-1}$) over 3 h or in several fractions at a higher dose rate (25 cGy min$^{-1}$) over a longer period of time (2 days) gave similar early effects on the murine haemopoietic compartment. Furthermore, a month later there was still no significant difference between the two groups although slightly higher values were observed in the FTBI group.

The early effects after the end of radiation treatments (i.e. the first 48 h) were similar in both groups for all cells except for bone marrow cells. CFU-S and GM-CFC clonogenic cells were reduced immediately after radiation in both groups by 2.8 and 2.5 logs respectively. Differences in dose rates between the 2 schedules (4 cGy min$^{-1}$ versus 25 cGy min$^{-1}$) were not very large and therefore it is likely that they played a very minimal role, as previously shown in some authors (Frindel *et al.*, 1972; Glasgow *et al.*, 1983; Tarbell *et al.*, 1987; Travis *et al.*, 1985; Evans *et al.*, 1988). The similar early effects found in both groups suggest first, that fractionation did not play any role in the sparing of the CFU-S and GM-CFC clonogenic cells, and second that no significant repopulation took place during the fractionated TBI. These conclusions are further supported by the fact that between the two groups no significant differences in the numbers of CFU-S and GM-CFC were observed during the 1 month observation period.

The first day after irradiation, peripheral blood granulocyte numbers were slightly increased in both groups. This transient increase was probably due to a release of mature granulocytes from bone marrow as shown by Harris (1959) and Dutreix *et al.*, (1987). Differences in bone marrow cell numbers the first day after irradiation were probably due to different treatment durations between the two groups (3 h for the STBI groups and 36 h for the FTBI group); a longer treatment time allowing the elimination of doomed cells.

The pattern of repopulation of bone marrow cells, and CFU-S was similar in both groups and lead to a complete recovery 1 month later. This finding suggests that bone marrow clonogenic cells and possibly their microenvironment, thought to be critical in haemopoietic recovery (Hendry, 1985; Schofield & Dexter, 1982) were similarly affected by the two different radiation protocols. However, a much longer follow-up is warranted to determine damage caused to the microenvironment by the different radiation treatments.

Both irradiations induced the same changes in clonogenic cell differentiation resulting in an early increase in granulocyte colonies and a concomitant decrease in erythrocyte colonies as already reported by Frindel *et al.* (1980). The E/G ratios were significantly decreased in both groups, soon after irradiation with a return to normal values by the end of the second week (Table II).

GM-CFC clonogenic bone marrow cells in both groups showed a similar repopulation pattern except on day 7 when significantly different surviving fractions were observed. This finding might be due to an earlier start in either GM-CFC repopulation or CFU-S differentiation towards GM-CFC progenitors in the FTBI group. However, it cannot be excluded that the difference is purely artefactual since the analysis was performed 9 days after the start of the fractionated radiotherapy and only 8 days after the start of the single dose radiotherapy.

Repopulation of the peripheral blood granulocytes was similar in both groups with a slight overshoot by the end of the month for which there is no clear explanation. On the other hand lymphopenia was still present at the end of the observation period in both groups suggesting that both types of irradiation are equally immunosuppressive.

In conclusion, although the underlying mechanisms are complex and not fully understood, our study indicates that fractionated and single dose TBI have similar effects on the murine haemopoietic system. It suggests that both TBI regimens could be given to patients for bone marrow transplantation provided that radiation damage to the lung is not increased by either of the two schedules. However, these results should be corroborated with a larger range of doses for firm conclusions to be drawn and is the subject of an ongoing study.

The work was performed in the laboratory of Dr Frindel (Unité INSERM 250 Villejuif) and was supported by a grant no. 85 D 12 from Gustave-Roussy Institute. The authors wish to thank E. Frindel, M. Guigon, D. Dumenil and N. Blackett (Unité INSERM 250) for useful discussions, C. Lacout for her excellent technical help, L. Saint Ange and P. Frazee for their work in the preparation of the manuscript and C. Chimchirian for her excellent secretarial assistance.

### References

DUTREIX, J., GIRINSKY, T., COSSET, J.M. & 5 others (1987). Blood cell kinetics and total body irradiation. *Radiother. Oncol.*, **9**, 119.

EVANS, R.G., WHEATLEY, C.L. & NIELSEN, J.R. (1988). Modification of radiation induced damage to bone marrow stem cells by dose rate, dose fractionation and prior exposure to cytoxan as judged by the survival of CFUs: application to bone marrow transplantation (BMT). *Int. J. Radiat. Oncol. Biol. Phys.*, **14**, 491.

FRINDEL, E., DUMENIL, D. & SAINTENY, F. (1980). Role of pluripoietins in murine bone marrow stem cell differentiation. *Leukemia Res.*, **4**, 287.

FRINDEL, E., HAHN, G.M., ROBAGLIA, D. & TUBIANA. M. (1972). Responses of bone marrow and tumor cells to acute and protracted irradiation. *Cancer Res.*, **32**, 2096.

GLASGOW, G.P., BEETHAM, K.L. & MILL, W.B. (1983). Dose rate effects on the survival of normal hematopietic stem cells of Balb/c mice. *Int. J. Radiat. Oncol. Biol. Phys.*, **9**, 557.

GUYOTAT, D., DUTOU, L., EHRSAM, A., CAMPOS, L., ARCHIMBAUD, D. & FIERE, D. (1987). Graft rejection after T cell depleted marrow transplantation: role of fractionated irradiation. *Br. J. Haematol.*, **65**, 499.

HAGENBEEK, A. & MARTENS, A.C.M. (1981). The effect of fractionated versus unfractionated total body irradiation on the growth of the BN acute myelocytic leukemia. *Int. J. Radiat. Oncol. Biol. Phys.*, **7**, 1075.

HARRIS, P.F. (1959). The correlation between bone marrow activity and blood neutrophil levels from quantitative studies in irradiated guinea pigs. *Br. J. Exp. Pathol.*, **40**, 589.

HENDRY, J.H. (1985). The cellular basis of long term marrow injury after irradiation. *Radiother. Oncol.*, **3**, 331.

KREBS, J.S. & JONES, D.C.L. (1972). The LD50 and the survival of bone marrow colony forming cells in mice. Effect of rate of exposure to ionizing radiation. *Radiat. Res.*, **51**, 374.

PATTERSON, J., PRENTICE, H.G., BRENNER, M.K. & 21 others (1986). Graft rejection following HLA matched T lymphocyte depleted bone marrow transplantation. *Br. J. Haematol.*, **63**, 221.

PEACOCK, J.M., STEEL, G.G. & STEPHENS, T.C. (1986). Radiation dose rate dependent differences in cell kill and repopulation in murine bone marrow CFU-S and CFU-C. *Br. J. Cancer*, **53**, 171.

PETERS, L. (1980). Discussion: the radiobiological bases of TBI. *Int. J. Radiat. Oncol. Biol. Phys.*, **6**, 785.

PETERS, L.J., WITHERS, H.F., CUNDIFF, J.H. & DICKE, K.D. (1979). Radiobiological considerations in the use of total body irradiation for bone marrow transplantation. *Radiology*, **131**, 243.

PURO, E.A. & CLARK, G.M. (1972). The effect of exposure rate on animal lethality and spleen colony survival. *Radiat. Res.*, **52**, 115.

SCHOFIELD, R. & DEXTER, T.M. (1982). CFU-S repopulation after low dose whole body radiation. *Radiat. Res.*, **89**, 607.

SHERIDAN, J.W., & METCALF, D. (1973). A low molecular weight factor in lung conditioned medium stimulating granulocyte and monocyte colony formation *in vitro*. *J. Cell. Physiol.*, **81**, 11.

SONG, C.W., KIM, T.H., KHAN, M.F., KERSEY, J.H. & LEVITT, S.H. (1987). Radiobiological basis of total body irradiation with different dose rate and fractionation: repair capacity of hemopoietic cells. *Int. J. Radiat. Oncol. Biol. Phys.*, **7**, 1695.

TARBELL, N.J., AMATO, D.A., DOWN, J.D., MAUCH, P. & HELLMAN, S. (1987). Fractionation and dose rate effects in mice: a model for bone marrow transplantation in man. *Int. J. Radiat. Oncol. Biol. Phys.*, **13**, 1065.

TRAVIS, E.L., PETERS, L.J., MCNEILL, J., THAMES, M.D. & KAROLIS, C. (1985). Effect of dose rate on total body irradiation: lethality and pathologic findings. *Radiother. Oncol.*, **4**, 341.

WORTON, R., TILL, J.E. & MCCULLOCH, E.A. (1969). Physical separation of hemopoietic stem cells from cells forming colonies in culture. *J. Cell. Physiol.*, **74**, 171.